# Idiopathic Radiation Recall Dermatitis Developing Nine Months after Cessation of Cisplatin Therapy in Treatment of Squamous Cell Carcinoma of the Tonsil

**DOI:** 10.1155/2012/271801

**Published:** 2012-06-14

**Authors:** Stephen M. Melnyk, Kenneth F. More, Edward F. Miles

**Affiliations:** ^1^School of Medicine, Uniformed Services University of the Health Sciences, 4301 Jones Bridge Road, Bethesda, MD 20814, USA; ^2^Division of Hematology/Oncology, Department of Medicine, Naval Medical Center Portsmouth, 620 John Paul Jones Circle, Portsmouth, VA 23708, USA; ^3^Division of Radiation Oncology, Department of Radiology, Naval Medical Center Portsmouth, 620 John Paul Jones Circle, Portsmouth, VA 23708, USA

## Abstract

To report on a suspected case of idiopathic radiation recall dermatitis in an individual nine months after radiation and chemotherapy treatment of squamous cell carcinoma of the right tonsil. Radiation recall dermatitis is the development of a reaction in a previously irradiated area of skin after the administration of an aggravating medication. A review of the literature revealed several cases of radiation recall dermatitis that occur following radiation therapy and the institution of chemotherapy. Other medications have also been implicated in radiation recall dermatitis; however, this patient has not started any new medications since completion of his combined therapy. The patient developed this skin reaction in a distribution pattern identical to the area that received the highest radiation dose suggesting a possible link between radiation recall dermatitis and radiation dose. Radiation recall dermatitis is a reaction that is typically seen shortly after the reinstitution of chemotherapy during radiation therapy. This case illustrates that other medical etiologies are possible and suggests a relationship between radiation recall dermatitis and the total radiation dose an area receives.

## 1. Introduction

Radiation recall dermatitis is the development of a dermatologic reaction in a previously radiated area of skin after the administration of an aggravating medication. The first case of radiation recall dermatitis was reported in 1959 by D'Angio et al. who noticed the reaction following treatment with dactinomycin [1]. Here we present a case of idiopathic radiation recall dermatitis that developed nine months after concurrent radiation therapy and cisplatin chemotherapy.

## 2. Case Report

The patient is a 49-year-old Caucasian male that initially presented with a three-to-four month history of unilateral tonsillar swelling. Computer tomography of the neck revealed bilateral tonsillar enlargement, right greater than left, with a right internal jugular chain lymph node measuring 1.5 cm by 1.5 cm. The patient underwent a bilateral tonsillectomy without complications. Pathology of the left tonsil was benign, but the right tonsil contained a 2.4 cm, poorly differentiated, squamous cell carcinoma. The remainder of his staging work-up was negative, and he was staged with T2N1M0, Stage III invasive squamous cell carcinoma of the right tonsil.

Combined modality therapy with concurrent cisplatin chemotherapy and daily radiation therapy was initiated. The tumor bed and ipsilateral neck were treated with 200 centi-Gray (cGy) fractions to 7,000 cGy, while the contralateral neck received 170 cGy fractions to 5,600 cGy. The cisplatin therapy was administered at a dose of 100 mg per meter squared on days one, twenty-two and forty-three of his six weeks of radiation. The patient was able to complete his treatment as prescribed without the need for a treatment break. He developed grade II erythema within the radiation field on bilateral necks, dysgeusia, xerostomia, and mucositis symptoms but otherwise did well.

**Figure 1:**
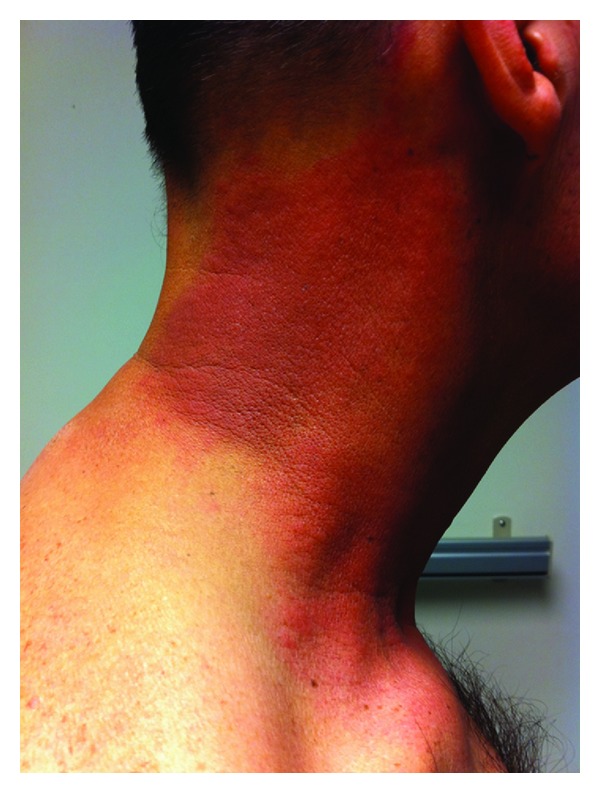
Lateral aspect of patient's right neck demonstrating affected area.

**Figure 2:**
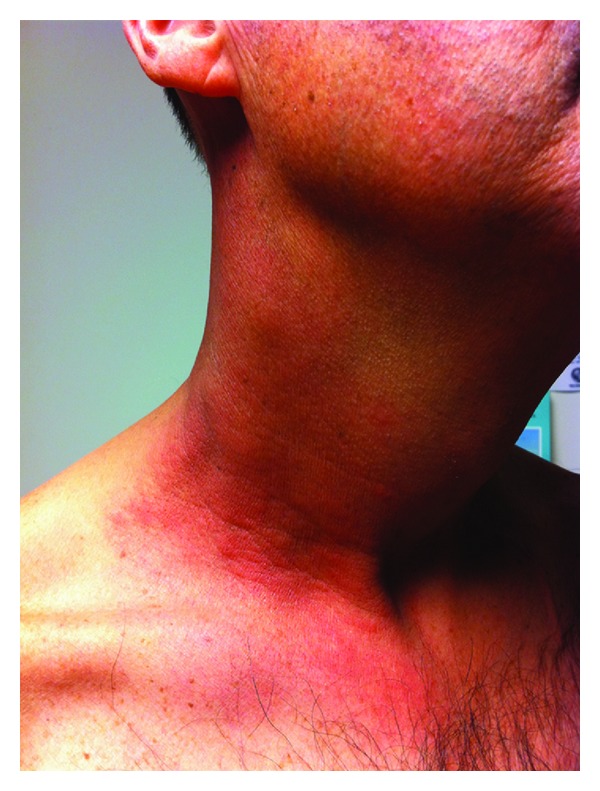
Anterolateral aspect of patient's right neck.

Following completion of his treatment, the patient was able to return to work and displayed regular improvement during his scheduled follow-up visits. The erythema that developed during his treatment had completely resolved and the patient regularly reported feeling well. Approximately nine months after his last radiation or chemotherapy treatment, the patient reported to the clinic for routine followup. He noted a twenty-four hour history of a tender, hot, confluent rash on his right neck that began at his clavicle and extended superiorly to the mandibular angle (Figures 1 and 2). He reported an oral temperature of 102 F; his temperature in the clinic was 101.2 F. He also experienced some myalgias and arthralgias but denied constitutional symptoms of nausea, vomiting, weight loss, drenching night sweats, and fatigue. He denied any respiratory symptoms, cough, hemoptysis, or bone pain. The patient had received no interval chemotherapy and had not started any new medications since his last visit. Radiation recall dermatitis was suspected in this patient given the distribution of the rash and presenting symptoms. Due to the warmth of the tissue and the fever, there was some concern for cellulitis as well, which prompted treatment with doxycycline. The patient was seen several months after this clinical encounter for routine followup and reported that his rash resolved in the five to six days following the initial presentation. He continued to report experiencing xerostomia and dysgeusia; he denied any recurrence of his rash.

## 3. Discussion

Radiation recall dermatitis is the development of a localized skin reaction following radiation therapy that can be induced by a variety of medications. Some of the medications implicated in this reaction include actinomycin [1], adriamycin [2], docetaxel [3], doxorubicin [4], gatifloxacin [5, 6], gemcitabine [7], interferon alpha-2b [8], levofloxacin [9], paclitaxel [10], pemetrexed [11], simvastatin [12], and tamoxifen [13] among others.

Of note in this case are both the distribution of the radiation recall dermatitis and the timing of the reaction. The patient received radiation along bilateral necks but only developed a skin reaction in the area of the ipsilateral neck that received the highest daily dosage and total dose during treatment. This suggests that the development of radiation recall dermatitis might be dependent on either the fraction size, total radiation dose, or both.

Radiation recall dermatitis typically develops within days of initiation of the offending agent. In a broad review of cases, Camidge and Price determined the median interval between radiation and chemotherapy was 39.5 days; however, the longest interval between drug administration and skin reaction was two months [14, 15]. In this patient, the reaction was seen nine months following the final dose of cisplatin suggesting that the window for developing the skin reaction is significantly longer than originally thought or another etiology is responsible in this case. During this nine-month period, the patient received no other cytotoxic, hormonal, or antimicrobial pharmacotherapy, nor did he change any soap, detergent, cologne, or skin care product.

## 4. Conclusion

Radiation recall dermatitis is an acute reaction that occurs in previously irradiated skin following the administration of an aggravating medication. The skin reaction typically develops within a few days of administration of the involved medication. We present a case of idiopathic radiation recall dermatitis that developed nine months after the cessation of chemotherapy with no new medications initiated during the interim. This case suggests that radiation recall dermatitis may have a more complex etiology than originally described. This case also implicates that the fraction size or total radiation dosage might also be important factors in the development of the dermatologic reaction.
